# Carnitine, Amino Acids, Vitamins, and Hematological Status in Children with Classical Phenylketonuria: A Case–Control Study

**DOI:** 10.3390/nu18152407

**Published:** 2026-07-23

**Authors:** Sezai Arslan, Esra Dişci

**Affiliations:** 1 Department of Inherited Metabolic Diseases, Erzurum City Hospital, Erzurum 25240, Türkiye; 2 Department of Pediatrics, Erzurum City Hospital, Erzurum 25240, Türkiye; doctor_esadi@hotmail.com

**Keywords:** phenylketonuria, carnitine, amino acids, vitamins, body mass index

## Abstract

**Background:** Classical phenylketonuria (PKU) requires the lifelong dietary restriction of phenylalanine-containing foods. In this study, we aimed to evaluate the effects of a phenylalanine-restricted diet on carnitine, amino acid, vitamin, and mineral status in children with classical PKU. **Methods:** This case–control study included 30 children with classical PKU and 30 age- and sex-matched healthy controls. Dietary adherence was categorized as good, moderate, or poor according to blood phenylalanine concentrations during the preceding year. Dried blood spot amino acid and acylcarnitine profiles combined with serum micronutrient and hematological parameters were analyzed. **Results:** No significant differences were observed between the PKU and control groups regarding tyrosine, free carnitine (C0), acetyl carnitine (C2), valine, or methionine concentrations (*p* > 0.05). Arginine levels were significantly lower in the PKU group than in controls (40.14 ± 29.89 vs. 48.25 ± 16.08 µmol/L, *p* = 0.008). Vitamin B12, folate, 25-hydroxyvitamin D, and mean corpuscular volume were significantly higher in patients with PKU (all *p* < 0.05). Dietary adherence exhibited a strong negative correlation with phenylalanine concentrations (r = −0.86, *p* < 0.001), a moderate negative correlation with the phenylalanine-to-tyrosine ratio (r = −0.56, *p* = 0.001), and a moderate negative correlation with body mass index (r = −0.55, *p* = 0.002). **Conclusions:** Children with classical PKU receiving long-term dietary treatment maintained adequate carnitine and micronutrient status. Reduced arginine concentrations observed in treated children with classical PKU warrant further investigation, although their clinical significance remains uncertain.

## 1. Introduction

Classical phenylketonuria (PKU) is a relatively rare, autosomal recessive condition affecting the metabolism of the amino acid phenylalanine (Phe); due to a deficiency in the phenylalanine hydroxylase (PAH) enzyme, the conversion of Phe to tyrosine (Tyr) is partially or completely inhibited [1,2,3]. Elevated Phe levels impair the LAT1 transporter, thereby reducing the transport of other neutral amino acids into the brain. Consequently, it has been demonstrated to induce reduced protein synthesis in the brain, decreased neurotransmitter synthesis (dopamine, serotonin, and noradrenaline), and myelination abnormalities [1,3,4]. The global prevalence of PKU ranges from approximately 1:10,000 to 1:15,000, with higher rates observed in countries such as Italy (1:2700), Türkiye (1:4370), Ireland (1:4500), and Iran (1:4698) [1,5].

In classical PKU, serum Phe levels are typically above 1200 μmol/L, and patients exhibit complete PAH enzyme deficiency [6]. In untreated classical PKU, clinical symptoms include profound intellectual disability, epilepsy, behavioral disturbances, microcephaly, and hypopigmentation of hair and skin resulting from impaired melanin synthesis derived from tyrosine [4,7].

In the dietary management of classical PKU, high-protein animal foods (such as meat, poultry, fish, and eggs), dairy products, grains (wheat, oats, barley, and rye), and legumes and nuts are prohibited, and a daily intake of Phe is limited to 200–400 mg (one gram of natural protein = 50 milligrams of Phe) [7]. Insufficient Phe intake is recognized to lead to loss of appetite, fatigue, hair loss, perineal dermatitis, and growth retardation [3]. Current guidelines recommend maintaining metabolic control throughout life [3,7]. Consequently, the diet of such patients consists of special amino acid formulations (Phe-free), low-protein flour/bread, vegetables, and fruit. The results of previous studies have indicated that individuals with PKU may exhibit deficiencies in iron, vitamin B12, zinc, vitamin D, selenium, and coenzyme Q10 in particular [3,8]. It is believed that non-compliance with the diet may lead to both hyperphenylalaninemia and vitamin deficiencies associated with inadequate and unbalanced nutrition.

Carnitine is a water-soluble molecule that promotes the transport of long-chain fatty acids into mitochondria and is essential for energy metabolism, with meat, poultry, fish, and dairy products serving as primary sources. It is also produced endogenously in the liver by the amino acids lysine and Met. Carnitine possesses anti-inflammatory, antioxidant, and insulin-sensitizing properties. Symptoms of carnitine insufficiency include hypoketotic hypoglycemia, cardiomyopathy, myopathy, and liver disease [9,10,11]. It has been demonstrated that carnitine intake is reduced in classic PKU cases, resulting in low serum carnitine levels [3,10,11].

Furthermore, the PKU diet has been shown to influence the levels of essential and semi-essential amino acids [12]. Tyr, in particular, becomes an essential amino acid in PKU cases because it cannot be synthesized from Phe, and it can impair the synthesis of dopaminergic/catecholaminergic neurotransmitters [1,2,3]. While individual micronutrients or amino acids in patients with PKU have been assessed in numerous studies, there is a paucity of data that concurrently provides insight into carnitine status, essential and semi-essential amino acids, vitamins, and hematological parameters in children with classical PKU, especially in populations utilizing modern protein substitute formulations. Moreover, data from Türkiye are limited. To address these gaps, we aimed to thoroughly assess these nutritional parameters in children with classical PKU in comparison to healthy controls.

## 2. Materials and Methods

### 2.1. Study Design and Setting

This case–control study was conducted between July 2025 and March 2026 and included 30 individuals aged 2–18 years with classical phenylketonuria (PKU) and 30 age- and sex-matched healthy controls.

The sample size estimation was based on the expected between-group difference in plasma arginine concentrations reported by Schulpis et al. [13]. Arginine was selected as the reference variable because comparable published data were available from a previous study evaluating amino acid profiles in children with classical phenylketonuria. The sample size was calculated using G*Power version 3.1 (Heinrich Heine University Düsseldorf, Düsseldorf, Germany). To detect a between-group difference of 8 units, corresponding to an effect size of d = 0.74, with 80% power and a two-sided α level of 0.05, a total of 60 participants, with 30 participants in each group, were required.

### 2.2. Participants and Eligibility Criteria

The diagnosis of classical PKU was confirmed by elevated blood phenylalanine concentrations and molecular analysis of the PAH gene demonstrating either homozygous or compound heterozygous pathogenic variants according to the American College of Medical Genetics and Genomics (ACMG) criteria. Consecutive eligible patients attending routine follow-up visits during the study period were invited to participate. All invited patients agreed to participate, and none declined enrollment.

Controls were recruited from children attending the general pediatric outpatient clinic for routine health examinations and had no known metabolic, chronic, or hematological disorders. We excluded participants who had received vitamin or mineral supplements during the previous six months, in addition to those who had acute infections or chronic systemic disorders.

### 2.3. Data Collection and Dietary Management

All blood samples were collected after an overnight fast and before the morning administration of the phenylalanine-free protein substitute. Venous blood samples were obtained from participants for hematological and biochemical analyses. The hemogram analysis was performed on the Mindray BC-6800 Plus fully automated hematology analyzer (Mindray Bio-Medical Electronics Co., Shenzhen, China). Serum ferritin, vitamin B12, folate, and 25-hydroxyvitamin D [25(OH)D] levels were measured using the immunoassay method with the Siemens Atellica Immunoassay Analyser (Siemens Healthineers, Erlangen, Germany). Serum zinc level was measured using the Siemens Atellica Biochemistry Analyser (Siemens Healthineers, Erlangen, Germany).

Dried blood spot amino acid profiles were analyzed using the LC-MS/MS method with the SCIEX 3500 Triple Quad system (SCIEX, Framingham, MA, USA); the dry blood acylcarnitine profile was analyzed using the LC-MS/MS method with the Shimadzu LCMS-8030 system (Shimadzu Corporation, Kyoto, Japan).

Dietary management was individualized according to age, body weight, growth requirements, and metabolic control. In general, patients received age-appropriate energy intake combined with restricted natural protein and phenylalanine-free protein substitutes to meet daily protein requirements while providing approximately 200–400 mg/day of essential phenylalanine from natural foods. Phenylalanine intake was adjusted during follow-up according to serial blood phenylalanine concentrations, growth parameters, and clinical evaluation. However, detailed quantitative dietary records, total energy intake, natural protein intake, and actual daily consumption of protein substitutes were not systematically recorded; therefore, these data were not included in the statistical analyses.

Dietary adherence was evaluated according to metabolic control based on blood phenylalanine (Phe) concentrations obtained during the preceding year. Target blood Phe concentrations were defined as 120–360 μmol/L for children younger than 12 years and 120–600 μmol/L for those aged 12 years and older, in accordance with current guidelines [3]. Patients were classified as displaying good adherence (0–2 measurements outside the target range), moderate adherence (3–4 measurements outside the target range), or poor adherence (≥5 measurements outside the target range). This classification reflected metabolic control and did not specifically assess adherence to phenylalanine-free protein substitute consumption. In addition, the type of medical protein substitute used (powdered or ready-to-drink formulations, including sachet forms) was recorded, and its association with dietary adherence was analyzed. The nutritional composition of the protein substitutes is summarized in Supplementary Table S1.

### 2.4. Statistical Analysis

Statistical analyses were performed using IBM SPSS Statistics for Windows, version 20.0 (IBM Corp., Armonk, NY, USA). Data are presented as mean ± standard deviation, median (minimum–maximum), count, and percentage, as appropriate. The normality of continuous variables was evaluated using the Shapiro–Wilk test, Kolmogorov–Smirnov test, Q–Q plots, and assessments of skewness and kurtosis. Comparisons between two independent groups were conducted using the independent samples test for normally distributed variables and the Mann–Whitney U test for non-normally distributed variables. For comparisons of continuous variables among more than two independent groups, one-way analysis of variance (ANOVA) was utilized when the normality assumption was satisfied, whereas the Kruskal–Wallis test was employed when the assumption was violated. Following ANOVA, post hoc analyses were performed using Tukey’s test when variances were homogeneous and Tamhane’s T2 test for non-homogeneous variances. For the Kruskal–Wallis test, post hoc comparisons were conducted using the Kruskal–Wallis one-way ANOVA (k samples) procedure. To control for age as a potential confounder, analysis of covariance (ANCOVA) was performed, with protein substitute formulation (powder vs. ready-to-drink) as the fixed factor and age as the covariate. For the comparison of categorical variables in 2 × 2 contingency tables, Pearson’s chi-square test was used when expected cell counts exceeded 5, Yates’ continuity-corrected chi-square test was applied when expected counts were between 3 and 5, and Fisher’s exact test was utilized when expected counts were below 3. For categorical comparisons involving tables larger than 2 × 2, Pearson’s chi-square test was applied when expected counts were above 5, while the Fisher–Freeman–Halton test was used when expected counts were below 5.

## 3. Results

### 3.1. General Characteristics of the Cases Participating in This Study

Our study comprised a total of sixty participants, thirty of whom were assigned to the PKU group and thirty of whom were assigned to the control group. Analysis of the clinical characteristics of the study participants revealed that 46.7% (*n* = 14) exhibited poor dietary compliance, 10.0% (*n* = 3) showed moderate compliance, and 43.3% (*n* = 13) demonstrated good compliance. An assessment of the medical protein substitutes used revealed that 33.3% of patients (*n* = 10) used the powder form, while 66.7% (*n* = 20) used the ready-to-drink formula. The mean age of the children involved in the study was determined to be 8 ± 4 years. The analysis results revealed no statistically significant difference in age between the PKU (*n* = 30) and control (*n* = 30) groups (*p* = 0.6) (Table 1). The z-scores for height, weight, and body mass index (BMI) showed a similar distribution across the groups.

### 3.2. Comparison of Amino Acid, Vitamin, and Carnitine Levels Between Phenylketonuria and Control Groups

Upon examination of metabolic parameters, we found Phe levels in the PKU group (623.8 ± 447.8) to be significantly higher than those in the control group (60.9 ± 18.4) (*p* < 0.001). Similarly, the Phe/Tyr ratio was significantly higher in the PKU group (12.4 ± 14.7) (*p* < 0.001). Arg levels, however, were found to be statistically significantly lower in the PKU group (40.14 ± 29.89) compared with the control group (48.25 ± 16.08) (*p* = 0.008). In contrast, no significant difference was observed between the groups in terms of Tyr, C0, C2, Val, and Met levels (*p* > 0.05) (Table 2).

In addition, vitamin B12 (672 ± 314, *p* < 0.001), folate (18.1 ± 4.9, *p* < 0.001), and vitamin D (30.25 ± 10.09, *p* = 0.005) levels were found to be significantly higher in the PKU group compared with the control group. No significant difference was found between the groups in ferritin and zinc levels (*p* > 0.05). In terms of hematological parameters, it was found that MCV values in the PKU group (82.3 ± 5.1) were significantly higher than those in the control group (79.9 ± 3.3) (*p* = 0.027). No significant difference was detected in Hb and Hct values between the groups (*p* > 0.05) (Table 2).

When examining the status of the variables within the normal range in the groups, it was found that 93.3% (*n* = 28) of the patients in the PKU group had phenylalanine levels outside the normal range, while those of all individuals (100%) in the control group were within the normal range (*p* < 0.001). Similarly, the Phe/Tyr ratio was outside the normal range in 90.0% (*n* = 27) of the PKU group, while this ratio was 0% in the control group (*p* < 0.001). In terms of tyrosine levels, 20.0% of the PKU group fell outside the normal range, while all values in the control group were within normal limits (*p* = 0.024). Additionally, the vitamin B12 (36.7% vs. 3.3%; *p* = 0.001) and folate (37.9% vs. 3.3%; *p* = 0.001) levels outside the normal range were found to be significantly higher in the PKU group compared with the control group. In terms of vitamin D levels, it was observed that 89.7% of the PKU group had levels within the normal range, with this rate being 66.7% in the control group (*p* = 0.033). No significant difference was found between the groups in terms of the distribution of valine, ferritin, and zinc levels according to the reference range (*p* > 0.05). Carnitine (C0, C2), arginine, and methionine levels were largely observed to be within normal limits in both groups (Table 3).

### 3.3. A Comparative Analysis of Amino Acid, Vitamin, and Carnitine Levels in Patients with Classic Phenylketonuria Using Powdered Versus Ready-to-Drink Formulations

When the demographic and biochemical parameters of patients using powdered and ready-to-drink formulations were compared, statistically significant differences were found between the groups in only a few variables. In terms of age, the ready-to-drink formulation group was significantly older (*p* = 0.01); this difference necessitated the use of age as a covariate in the analysis. Upon examination of the biochemical parameters, Arg levels were found to be statistically significantly higher in the ready-to-drink group (*p* = 0.03); however, this difference lost its statistical significance when the effect of age was controlled for (*p* = 0.06). Phe (*p* = 0.6); Tyr (*p* = 0.09); Phe/Tyr ratio (*p* = 0.3); C2 (*p* = 0.5) and carnitine C0 (*p* = 0.4) fractions; and Val (*p* = 0.1), Met (*p* = 0.1), ferritin (*p* = 0.8), zinc (*p* = 0.5), folate (*p* = 0.3), and vitamin D (*p* = 0.09) levels did not show statistically significant differences between the groups (*p* > 0.05). However, in the age-adjusted covariate analysis, vitamin B12 levels were found to be significantly higher in the powder formulation group (*p* = 0.02); in the unadjusted comparison, this difference was not significant (*p* = 0.5) (Table 4). There were no statistically significant differences between the groups in terms of Hb (*p* = 0.7), Hct (*p* = 0.3), MCV (*p* = 0.1), height (*p* = 0.4), weight (*p* = 0.3), and BMI (*p* = 0.7); these variables showed no statistically significant differences between the groups in either the unadjusted or the covariate-adjusted analyses (*p* > 0.05).

### 3.4. The Impact of Compliance with a Phenylalanine-Restricted Diet on Growth Parameters, Amino Acid, Carnitine, and Vitamin Levels in Individuals with Classical Phenylketonuria

A strong negative correlation was found between dietary compliance and Phe levels (r = −0.8, *p* < 0.001). Similarly, a moderate negative correlation was observed with the Phe/Tyr ratio (r = −0.5, *p* = 0.001). Furthermore, a moderate negative correlation was found between dietary compliance and BMI (r = −0.5, *p* = 0.002). In terms of Tyr (r = −0.06, *p* = 0.74), C_2_ (r = 0.07, *p* = 0.71), C_0_ (r = −0.1, *p* = 0.3), Arg (r = 0.1, *p* = 0.3), Val (r = −0.04, *p* = 0.81), Met (r = 0.0, *p* = 1.0), ferritin (r = 0.2, *p* = 0.2), B12 (r = 0.2, *p* = 0.2), zinc (r = −0.1, *p* = 0.4), folate (r = 0.01, *p* = 0.95), vitamin D (r = 0.31, *p* = 0.09), Hb (r = 0.07, *p* = 0.6), Hct (r = 0.1, *p* = 0.5), MCV (r = 0.1, *p* = 0.4), height (r = 0.1, *p* = 0.4), weight (r = −0.2, *p* = 0.1), and age (r = −0.2, *p* = 0.1), no statistically significant correlation was found with dietary compliance (Table 5). Plasma arginine concentrations did not differ significantly among patients with good, moderate, and poor dietary adherence (Kruskal–Wallis test, H = 0.99, *p* = 0.609).

## 4. Discussion

In the present study, we aimed to comprehensively evaluate carnitine status, amino acid profiles, micronutrient concentrations, and hematological parameters in children with classical PKU receiving long-term dietary treatment. Three principal findings emerged. First, most micronutrient and carnitine parameters were preserved. Second, vitamin B12, folate, and vitamin D concentrations were higher in the PKU group. Third, plasma arginine concentrations were lower than those of healthy controls.

Current guidelines recommend lifelong treatment for individuals with PKU [3]. In some patients, successfully achieving complete adherence to the target Phe levels in a Phe-restricted diet may be challenging due to factors including the social environment, palatability issues related to Phe-free amino acid formulations, and limited access to low-protein products. Our findings revealed that 43.3% of the PKU cohort (*n* = 13) demonstrated good dietary adherence. Several factors may explain the observed dietary non-adherence.

In the PKU diet, patients’ daily protein requirements are met using protein substitutes that do not contain Phe. At present, these substitutes are available in various formulations—such as powder, ready-to-drink, and sachet—for different age groups. Overall, 66.7% of patients used ready-to-drink formulations. No significant differences were identified in Hb, vitamins, amino acids, or carnitine levels when comparing the two formulation groups (powder and ready-to-drink).

In several studies from different countries, researchers have examined vitamin concentrations (including B12, folate, and vitamin D) and trace elements (such as zinc, selenium, and copper) in individuals with classical PKU, characterized by their restriction of high-quality animal proteins. A meta-analysis performed in 2024 indicated that although folate levels may be increased in individuals with PKU, vitamin B12 and 25-hydroxyvitamin D levels do not vary from those in the general population [14]. Research in Türkiye involving 112 individuals with classic PKU revealed elevated levels of vitamin B12 and folate, with 53.6% of patients exhibiting vitamin D insufficiency [15]. In our study, higher vitamin B12, folate, and vitamin D concentrations observed in the PKU group may be related to the use of micronutrient-fortified protein substitutes, although this hypothesis could not be directly evaluated because quantitative dietary intake data were not collected. However, this finding should be interpreted with caution because of the small subgroup sizes and the exploratory nature of the subgroup analyses.

The results of a study conducted in the Netherlands indicated that 14% of PKU patients adhering to a dietary regimen exhibited deficient zinc levels [16]. Our findings indicated no significant differences in zinc levels between the PKU group and the control group. Moreover, although our patients’ Hb and Hct levels were similar to those of the control group, their MCV values were slightly but significantly higher. Although higher MCV values were observed in the PKU group, the underlying mechanism remains uncertain, and the finding should be interpreted cautiously.

In healthy individuals, Arg is acquired through dietary sources and synthesized de novo to establish the Arg pool. It is considered a semi-essential amino acid during early childhood. Arg is metabolized into nitric oxide, urea, creatine, proline, glutamate, GABA, polyamines, and spermine through the action of numerous enzymes. It also contributes to protein synthesis [17,18]. The findings of a study involving 37 PKU patients exhibiting inadequate dietary compliance revealed significantly decreased Arg levels in comparison to a control group of 50 individuals [13]. In another study, researchers identified decreased Arg levels in 19% of 60 PKU individuals aged 1 to 39 years [16]. Although plasma arginine concentrations were lower in children with classical PKU than in healthy controls, no significant differences were observed among the good-, moderate-, and poor-adherence groups. This finding suggests that metabolic control based solely on blood phenylalanine concentrations may not adequately reflect arginine status. As adherence to phenylalanine-free protein substitute consumption was not directly assessed, the potential contribution of protein substitute intake to plasma arginine concentrations could not be determined.

Recent evidence suggests that both amino acid-based and glycomacropeptide-based protein substitutes can generally provide adequate nutritional support and maintain satisfactory micronutrient status when consumed regularly. Moreover, glycomacropeptide-based products have been associated with improved palatability, fewer gastrointestinal side effects, and higher acceptability, factors that may enhance long-term adherence to dietary treatment in patients with phenylketonuria [19,20]. Although glycomacropeptide-based protein substitutes may offer advantages in terms of acceptability and adherence, their limited availability in Türkiye currently precludes their widespread use, and amino acid-based protein substitutes remain the mainstay of dietary treatment for most patients with classical phenylketonuria.

A meta-analysis of individuals with classical PKU revealed a higher BMI compared with healthy controls [21]. An elevated BMI was reported to be two to three times more prevalent among adult female PKU patients. This finding may be linked to dietary consumption, psychological condition, social environment, and lifestyle factors [22]. In a study involving 46 PKU patients aged 13–17, researchers found no significant difference in obesity prevalence compared with the age-matched cohort [23]. Our investigation revealed a significant negative link between adherence to a Phe-restricted diet and blood Phe levels, a moderate correlation with the Phe/Tyr ratio, and a moderate negative correlation with BMI. Better dietary adherence was associated with lower BMI values; however, owing to the case–control study design, no causal inference regarding obesity prevention can be made.

This study has several limitations that must be acknowledged. The relatively small sample size may have limited statistical power to detect differences in some amino acid and carnitine parameters and increased the possibility of type II error; therefore, non-significant findings should not be interpreted as evidence of equivalence between groups. Furthermore, quantitative dietary intake and adherence to protein substitute consumption were not directly assessed, precluding causal inferences regarding the relationship between dietary treatment and biochemical findings. Lastly, biochemical measurements were obtained at a single time point and may not fully reflect long-term nutritional status. In addition, multiple biochemical parameters were compared without applying a formal correction for multiple testing because the analyses were primarily exploratory. Therefore, statistically significant findings, particularly those from secondary comparisons, should be interpreted as hypothesis-generating and confirmed in larger prospective studies, as the possibility of false-positive findings cannot be excluded.

Our findings provide further evidence that children with classical phenylketonuria receiving long-term dietary treatment can achieve satisfactory carnitine and micronutrient status. The lower arginine concentrations observed in the PKU group may represent a potential indicator of nutritional adequacy and dietary management; however, their clinical relevance has not yet been established. Future multicenter prospective studies incorporating detailed dietary assessments are needed to determine whether plasma arginine measurements offer additional value in the nutritional follow-up of patients with classical PKU. Treatment practices, dietary counseling strategies, and follow-up protocols may differ across metabolic centers; therefore, caution is warranted when extrapolating our findings to other populations.

## 5. Conclusions

In conclusion, children with classical PKU receiving long-term dietary treatment demonstrated preserved carnitine status and adequate micronutrient profiles. However, arginine concentrations were significantly lower than those of healthy controls. Further multicenter studies with larger sample sizes are required to determine the clinical significance of reduced arginine concentrations in PKU.

## Figures and Tables

**Table 1:** The study groups’ general characteristics.

		N	N%
Group	PKU	30	50.00%
	Control	30	50.00%
Diet compliance	Poor	14	46.70%
	Moderate	3	10.00%
	Good	13	43.30%
Phenylalanine-free protein substitute	Powder formulation	10	33.30%
	Ready-to-drink formulation	20	66.70%
		Mean ± SD	Median (Min–Max)
Age (Year)		8 ± 4	7 (2–17)

**Table 2:** A comparative analysis of amino acid profiles, Phe/Tyr ratios, free and acetyl carnitine concentrations, and vitamin levels between the classical phenylketonuria cohort and the control group.

	PKU (*n* = 30)		Control (*n* = 30)		
	Mean ± SD	Med (Min–Max)	Mean ± SD	Med (Min–Max)	*p*
Age (year)	8 ± 4	7 (2–17)	8 ± 4	6 (2–16)	0.63 ^&^
Phenylalanine (16–120 µmol/L) *	623.8 ± 447.8	536.0 (77–1823)	60.9 ± 18.4	56.1 (33.1–115.5)	**<0.001 ^&^**
Tyrosine (32–275 µmol/L)	86.8 ± 75.6	50.4(24.7–312)	80.6 ± 33.6	71.4 (33.3–209.9)	0.18 ^&^
Phe/Tyr (0.2–2)	12.4 ± 14.7	7.12 (0.96–54.09)	0.8 ± 0.2	0.7 (0.3–1.5)	**<0.001 ^&^**
Carnitine C2 (3.2–78 µmol/L)	19.1 ± 7.7	17.9 (9.98–42.2)	17.04 ± 5.40	16.02 (7.82–28)	0.38 ^&^
Carnitine C0 (15–60 µmol/L)	35.8 ± 8.6	36.1 (20.9–50)	32.1 ± 5.7	31.6 (23.6–44.9)	0.06 ^$^
Arginine (10–130 µmol/L)	40.1 ± 29.89	31.4 (10.1–121.6)	48.25 ± 16.08	47.45 (19.4–80.2)	**0.008 ^&^**
Valine (52–234 µmol/L)	148.5 ± 54.13	145.98 (61.8–257)	152.79 ± 56.27	159.2 (55.1–272.1)	0.76 ^$^
Methionine (6–58.8 µmol/L)	22.08 ± 9.32	21.6 (10.05–47)	23.03 ± 8.56	21.9 (10.7–47.4)	0.56 ^&^
Ferritin (22–322 ng/mL)	45.1 ± 58.4	32.1 (8.7–337)	29.4 ± 18.3	29.5 (4.8–78)	0.24 ^&^
Vitamin B12 (211–911 pg/mL)	672 ± 314	553 (293–1219)	371 ± 103	362 (156–644)	**<0.001 ^&^**
Zinc (60–120 µg/dL)	73 ± 23	71 (40–153)	70 ± 13	73 (39–87)	0.81 ^&^
Folate (5–20 ng/mL)	18.1 ± 4.9	18.7 (6.3–24)	11.4 ± 4.1	10.6 (5.5–22)	**<0.001 ^&^**
Vitamin D (20–100 ng/mL)	30.25 ± 10.09	30.4 (14.9–52.3)	23.06 ± 8.98	23 (8.6–52)	**0.005 ^$^**
Hb g/L	13.9 ± 1.0	13.8 (11.1–17.5)	13.5 ± 1.2	13.7 (11–16)	0.14 ^&^
Hct %	41.3 ± 3.0	41.1 (34–52.3)	41.1 ± 3.1	41 (36–48)	0.72 ^&^
MCV fL	82.3 ± 5.1	81.4 (66.3–92.4)	79.9 ± 3.3	80.2 (73.3–87.5)	**0.02 ^&^**

^$^: Independent samples *t*-test, ^&^: Mann–Whitney U test. * The values in brackets indicate the variable’s normal limits. Phe: phenylalanine; Tyr: tyrosine; Hb: hemoglobin; Hct %: hematocrit; MCV: mean corpuscular volume. Bold values indicate statistically significant differences (*p* < 0.05).

**Table 3:** Distribution of biochemical parameters according to reference ranges in children with classical phenylketonuria and healthy controls.

		PKU		Control			
		*n*	%	*n*	%	χ^2^	*p*
Phenylalanine	Within reference interval	2	6.70%	29	100.00%	51.514	<0.001
	Outside	28	93.30%	0	0.00%		
Tyrosine	Within reference interval	24	80.00%	29	100.00%		0.024
	Outside	6	20.00%	0	0.00%		
Phe/Tyr ratio	Within reference interval	3	10.00%	30	100.00%	49.091	<0.001
	Outside	27	90.00%	0	0.00%		
Acetylcarnitine C2 *	Within reference interval	28	100.00%	30	100.00%	NA	NA
	Outside	0	0.00%	0	0.00%		
Free carnitine C0 *	Within reference interval	28	100.00%	30	100.00%	NA	NA
	Outside	0	0.00%	0	0.00%		
Arginine *	Within reference interval	28	100.00%	30	100.00%	NA	NA
	Outside	0	0.00%	0	0.00%		
Valine *	Within reference interval	27	96.40%	28	96.60%		1.000
	Outside	1	3.60%	1	3.40%		
Methionine *	Within reference interval	28	100.00%	29	100.00%	NA	NA
	Outside	0	0.00%	0	0.00%		
Ferritin	Within reference interval	18	60.00%	17	56.70%	0.069	0.793
	Outside	12	40.00%	13	43.30%		
Vitamin B12	Within reference interval	19	63.30%	29	96.70%	10.417	0.001
	Outside	11	36.70%	1	3.30%		
Zinc *	Within reference interval	20	71.40%	25	83.30%	1.180	0.277
	Outside	8	28.60%	5	16.70%		
Folate *	Within reference interval	18	62.10%	29	96.70%	10.894	0.001
	Outside	11	37.90%	1	3.30%		
25-Hydroxy vitamin D *	Within reference interval	26	89.70%	20	66.70%	4.536	0.033
	Outside	3	10.30%	10	33.30%		
Dietary adherence	Poor	14	46.70%	0	0.00%	NA	NA
	Moderate	3	10.00%	0	0.00%		
	Good	13	43.30%	0	0.00%		
Protein substitute formulation	Powder	10	33.30%	0	0.00%	NA	NA
	Ready-to-drink	20	66.70%	0	0.00%		

NA: Not applicable, *: The denominators vary across variables because some biochemical measurements could not be completed owing to insufficient sample volume or unavailable specimens.

**Table 4:** Comparison of variables between the powder and ready-to-drink formulation groups.

	Powder Formulation		Ready-to-Drink Formulation			
Variables	Mean ± SD	Med (Min–Max)	Mean ± SD	Med (Min–Max)	*p*	*p*-Value from the Age-Adjusted Covariance Analysis
Age (year)	6 ± 3	6 (2–13)	9 ± 4	10 (3–17)	0.01 ^$^	
Phenylalanine (16–120 µmol/L)	582.5 ± 458.36	425 (86.8–1614)	644.4 ± 453.11	610.40 (77–1823)	0.69 ^&^	0.49
Tyrosine (32–275 µmol/L)	51.2 ± 25.7	44.3 (24.7–98.7)	104.6 ± 86.08	77.10 (24.97–312)	0.09 ^&^	0.38
Phe/Tyr ratio (0.2–2)	15.2 ± 16.8	7.2 (2.73–54.09)	11.09 ± 13.81	6.3 (0.9–53.7)	0.37 ^&^	0.42
Carnitine C2 (3.2–78 µmol/L)	21.4 ± 12.7	17.7 (9.98–42.2)	18.2 ± 4.7	17.9 (10.7–26.6)	0.51 ^$^	0.27
Carnitine C0 (15–60 µmol/L)	33.90 ± 10.01	33.2 (20.9–50)	36.6 ± 8.2	36.6 (22–49.7)	0.46 ^$^	0.20
Arginine (10–130 µmol/L)	22.85 ± 12.08	22.4 (10.1–41.2)	47.06 ± 32.2	32.35 (12.9–121.6)	0.03 ^&^	0.06
Valine (52–234 µmol/L)	124.7 ± 42.3	125.8 (61.8–187.7)	157.93 ± 56.3	161.9 (69.5–257)	0.14 ^$^	0.75
Methionine (6–58.8 µmol/L)	18.4 ± 5.4	19 (10–25.5)	23.5 ± 10.2	22.8 (10.4–47)	0.19 ^$^	0.65
Ferritin (22–322 ng/mL)	36.9 ± 24.5	30.6 (11.6–88.9)	49.2 ± 69.8	32.5 (8.7–337)	0.84 ^&^	0.48
Vitamin B12 (211–911 pg/mL)	743 ± 351	901 (293–1184)	637 ± 297	525 (309–1219)	0.50 ^&^	0.02
Zinc (60–120 µg/dL)	79 ± 31	75 (42–153)	70 ± 17	71 (40–121)	0.53 ^&^	0.55
Folate (5–20 ng/mL)	19.3 ± 4.2	20.1 (10.8–24)	17.4 ± 5.2	17.9 (6.3–24)	0.35 ^$^	0.12
Vitamin D (20–100 ng/mL)	36.2 ± 13.5	42.4 (14.9–52.3)	27.5 ± 6.9	29.8 (15.2–41.1)	0.09 ^$^	0.08

^$^: Independent samples *t*-test; ^&^: Mann–Whitney U test.

**Table 5:** Correlation of diet compliance with variables.

		Phe	Tyr	Phe/Tyr	Carnitine C2	Carnitine C0	Arginine	Valine	Met	Ferritin	Vitamin B12
Diet comp.	r	−0.862 **	−0.06	−0.564 **	0.07	−0.17	0.17	−0.04	0.00	0.22	0.21
	*p*	**0.000**	0.747	**0.001**	0.71	0.36	0.36	0.81	1.00	0.22	0.26
	N	30	30	30	28	28	28	28	28	30	30
		Age (Year)	Hgb g/L	Hct %	MCV fL	Height	Weight	BMI	Zinc	Folate	Vitamin D
Diet comp.	r	−0.25	0.07	0.10	0.13	0.14	−0.25	−0.546 **	−0.16	0.01	0.31
	*p*	0.18	0.68	0.56	0.48	0.45	0.17	**0.002**	0.40	0.95	0.09
	N	30	30	30	30	30	30	30	28	29	29

Reference ranges for laboratory parameters were as follows: Phenylalanine, 16–120 µmol/L; tyrosine, 32–275 µmol/L; Phe/Tyr ratio, 0.2–2.0; free carnitine (C0), 15–60 µmol/L; acetyl carnitine (C2), 3.2–78 µmol/L; arginine, 10–130 µmol/L; valine, 52–234 µmol/L; methionine, 6–58.8 µmol/L; ferritin, 32–322 ng/mL; vitamin B12, 211–911 pg/mL; zinc, 60–120 µg/dL; folate, 5–20 ng/mL; and 25-hydroxyvitamin D, 20–100 ng/mL. ** Diet comp.: Diet compliance; Phe: phenylalanine; Tyr: tyrosine; Met: methionine; Hgb: hemoglobin; Hct %: hematocrit; MCV: mean corpuscular volume; BMI: body mass index. Bold values indicate statistically significant differences (*p* < 0.05).

## Data Availability

The data that support the findings of this study are available upon request from the corresponding author.

## Supplementary Materials

The following supporting information can be downloaded at: https://www.mdpi.com/article/10.3390/nu18152407/s1. Table S1. Nutritional Composition of Protein Substitutes Used by Participants with Classical Phenylketonuria.

## Abbreviations

PKU	Phenylketonuria
Phe	Phenylalanine
Tyr	Tyrosine
C0	Free carnitine
C2	Acetyl carnitine
Arg	Arginine
Val	Valine
Met	Methionine
BMI	Body mass index
