# Supplementary material for: Carnitine, Amino Acids, Vitamins, and Hematological Status in Children with Classical Phenylketonuria: A Case–Control Study
Source: Nutrients. 2026 Jul 23;18(15):2407. doi: 10.3390/nu18152407 (PMC13468011; doi:10.3390/nu18152407)
Supplement: Supplementary file 1 [file nutrients-18-02407-s001.zip › nutrients-4392864-supplementary.pdf]

Table S1. Nutritional Composition of Protein Substitutes Used by Participants with Classical Phenylketonuria.

Formula	Manufacturer	Form	n	Protein Equivalent†	Vitamin B12‡ (µg)	Folate‡ (µg)	Vitamin D‡ (µg)	Carnitine‡ (mg)	Iron‡ (mg)	Zinc‡ (mg)
PKU Anamix	Nutricia	Powder	2	13.1 g/100 g	1.2	55	11.2	10.1	7.9	5.6
PKU 2 Prima	Nutricia	Powder	5	60 g/100 g	4.5	230	42	90	27	19.8
PKU 2 Secunda	Nutricia	Powder	1	70 g/100 g	4.2	253	35	105	22.4	20.0
Comida PKU B	ComidaMed	Powder	2	73 g/100 g	4.5	375	23	167	34	23.0
PKU Lophlex	Nutricia	Ready-to-drink	9	20 g/serving	1.76	120	8.0	12.8	5.2	3.8
PKU Gel	Vitaflo	Ready-to-drink	1	10 g/serving	0.48	50	3.5	11.0	3.4	2.6
PKU Express	Vitaflo	Ready-to-drink	1	15 g/serving	1.2	75	7.5	16.0	5.5	4.3
PKU Cooler	Vitaflo	Ready-to-drink	9	15 g/serving	1.2	75	7.5	ND	5.5	4.2
**Total**	—	—	**30**	—	—	—	—	—	—	—

† Protein equivalent values are expressed per 100 g of product for powdered formulations and per serving unit for ready-to-drink formulations.

‡ Nutrient contents were obtained from manufacturers' product information sheets available during the study period.

ND: not declared in the manufacturer's product specifications.
